# Second primary malignancies in thyroid cancer patients

**DOI:** 10.1038/sj.bjc.6601319

**Published:** 2003-10-28

**Authors:** C Rubino, F de Vathaire, M E Dottorini, P Hall, C Schvartz, J E Couette, M G Dondon, M T Abbas, C Langlois, M Schlumberger

**Affiliations:** 1 Unite INSERM XUR521, Gustave Roussy Institute, 39 rue Camille Desmoulins, Villejuif 94 805, France; 2 Nuclear Medicine Department, Ospedale Civile di Legnano, via Candiani 2, Legnano (Mi) I20025, Italy; 3 Department of Medical Epidemiology, Karolinska Institute, Berzelius Vag 15 c, Stockholm 17177, Sweden; 4 Nuclear Medicine Department, Jean Godinot Institute, 1 rue du Général Kœnig, Reims 51056, France; 5 Nuclear Medicine Department, François Baclesse Institute, route de Lion-sur-Mer, Caen 14076, France; 6 Nuclear Medicine Department, Gustave Roussy Institute, 39 rue Camille Desmoulins, Villejuif 94 805, France

**Keywords:** second primary malignancies, thyroid cancer, leukaemia, colorectal cancer, radioiodine, pooled analysis

## Abstract

The late health effects associated with radioiodine (^131^I) given as treatment for thyroid cancer are difficult to assess since the number of thyroid cancer patients treated at each centre is limited. The risk of second primary malignancies (SPMs) was evaluated in a European cohort of thyroid cancer patients. A common database was obtained by pooling the 2-year survivors of the three major Swedish, Italian, and French cohorts of papillary and follicular thyroid cancer patients. A time-dependent analysis using external comparison was performed. The study concerned 6841 thyroid cancer patients, diagnosed during the period 1934–1995, at a mean age of 44 years. In all, 17% were treated with external radiotherapy and 62% received ^131^I. In total, 576 patients were diagnosed with a SPM. Compared to the general population of each of the three countries, an overall significantly increased risk of SPM of 27% (95% CI: 15–40) was seen in the European cohort. An increased risk of both solid tumours and leukaemias was found with increasing cumulative activity of ^131^I administered, with an excess absolute risk of 14.4 solid cancers and of 0.8 leukaemias per GBq of ^131^I and 10^5^ person-years of follow-up. A relationship was found between ^131^I administration and occurrence of bone and soft tissue, colorectal, and salivary gland cancers. These results strongly highlight the necessity to delineate the indications of ^131^I treatment in thyroid cancer patients in order to restrict its use to patients in whom clinical benefits are expected.

Radioiodine (^131^I) has been used for over half a century for diagnostic purposes and for treating patients with hyperthyroidism and papillary or follicular thyroid carcinoma. The radiation dose delivered by radioiodine to nonthyroidal tissues is relatively low (International Commission on Radiological Protection (ICRP) 1988) and no increased risk of second primary malignancies (SPMs) linked to ^131^I was found in adult patients examined with ^131^I (Hall *et al*, 1996) or treated with ^131^I for hyperthyroidism (Ron *et al*, 1998; Franklyn *et al*, 1999). Much higher activities of ^131^I are used to treat thyroid cancer patients, resulting in significant radiation exposure. However, the late health effects associated with ^131^I given as treatment for thyroid cancer are difficult to assess since the number of thyroid cancer patients treated at each centre is comparatively small. To date, the impact of ^131^I on the occurrence of SPM in thyroid cancer patients has been studied in only three large cohorts (Hall *et al*, 1991; Dottorini *et al*, 1995; de Vathaire *et al*, 1997). In an effort to obtain a more accurate quantification of the overall risk of SPM, a pooled analysis of these three cohorts was performed and follow-up data were extended beyond the original publications.

The aim of the present study was to evaluate the risk of second cancer and leukaemia in a cohort of nearly 7000 Swedish, Italian, and French patients with papillary or follicular thyroid cancer, and to distinguish any pattern of risk related to exposure to internal radiation therapy given either alone or in association with external beam radiation therapy.

## PATIENTS AND METHODS

### Patients

A common database was obtained by pooling patients with papillary or follicular thyroid cancer of three major European cohorts. Patients with a malignancy prior to thyroid cancer, with a second malignancy within the first 2 years of follow-up, or dying within 2 years of diagnosis were excluded. The treatment modalities in each cohort have been described previously (Hall *et al*, 1991; Dottorini *et al*, 1995; de Vathaire *et al*, 1997).

The Swedish cohort consisted of patients initially treated between 1951 and 1977 at the Departments of Oncology of the six university hospitals in Sweden (Hall *et al*, 1991). The Italian cohort included patients initially treated between 1958 and 1995 in the Nuclear Medicine Department of the General Hospital in Busto Arsizio (Dottorini *et al*, 1995). The French cohort included patients initially treated between 1934 and 1995 in the Cancer Centres of Villejuif, Reims, and Caen (de Vathaire *et al*, 1997).

The end point of the study was fixed at 31 December 1997 and not later because, in the absence of recurrent disease, medical surveillance became less frequent after the first 5 years of follow-up.

The follow-up of patients of the Swedish cohort was updated through the Swedish Cancer Registry, and the follow-up of patients of the other two cohorts was updated through the medical records of each institution. Additionally, for each ^131^I administration, the date and activity were recorded in order to perform a time-dependent analysis (see Statistical analysis).

Compared to the previous reports, the Swedish cohort concerned the same patients but with a mean follow-up extended for 5 years. In the Italian cohort, patients initially treated between 1991 and 1995 were also included, as well as patients followed for less than 3 years who were previously excluded; patients who received external radiotherapy at the time of initial treatment were excluded, because technical parameters needed for dose calculation could not be obtained. In the French cohort, patients initially treated between 1992 and 1995 and patients initially treated by external radiotherapy were also included.

### Statistical analysis

A time-dependent analysis using external comparison was performed. Patients were considered at risk for second cancer during the period of time beginning 2 years after thyroid cancer diagnosis until any of the following four events: (1) 31 December 1997; (2) occurrence of an SPM; (3) death; (4) last visit to the ‘Cancer Centre’. Additionally, the occurrence of a third malignancy was considered in the site-by-site analysis only if it occurred during the first 2 years after a second malignant neoplasm. Malignancies that occurred later were excluded from the analysis since it was not possible to separate the effects of the thyroid cancer and SPM treatments.

Histological diagnosis was obtained for all second primaries and this allowed to exclude the possibility of metastases, especially in the lungs, bones, and brain.

The expected numbers of SPM were calculated by multiplying the gender, age, and calendar-year-specific person-years at risk with the corresponding incidence rates in each country. The data of the Swedish Cancer Registry (Cancer Incidence in Sweden, 2000) were used as reference rates for the Swedish cohort. Data of the registry of Varese, Lombardy, were used as reference rates for the patients included in the Italian cohort (Muir *et al*, 1987; Parkin *et al*, 1992, 1997) and for the 229 patients of the French cohort who came from Italy. The reference rates for the French patients were estimations of cancer incidences in France during the period 1975–1995 (Menegoz and Cherié-Challine, 2000). For sites not included in this estimation, two other data sets were used, covering the periods 1978–1982 (Benhamou *et al*, 1990) and 1983–1987 (de Vathaire *et al*, 1996). As no incidence rate was available before 1958 for Sweden, before 1976 for Italy, and before 1975 for France, the expected numbers of malignancies before these dates were calculated with the incidence rates of the following nearest period of time for each country. This approximation concerned only 6% of the follow-up.

The observed number of cancers was assumed to follow a Poisson distribution (Breslow and Day, 1987) and standardised incidence ratios (SIRs) were calculated as the ratio of observed to expected numbers. As age, gender, and calendar period were taken into account in the calculation of the expected numbers, the modelling of the SIRs was stratified only on country.

The exposure to external radiotherapy was considered as a binary variable. All ^131^I treatment courses administered up to 2 years before the end of the follow-up were taken into account. The ^131^I activity administered was analysed as a time-dependent variable: a patient, at a given time, was considered to be exposed to a risk of SPM related only to the cumulative activity administered previously. In this time-dependent analysis, the risk of SPM at a given calendar period, gender, and attained age was modelled as a function of the expected number of SPMs from the reference rates, and of the cumulative ^131^I activity administered 2 years or more before.

In order to estimate the risk of SPM per administered activity, excess relative risk (ERR) per GBq and excess absolute risk (EAR) per GBq and 100 000 person-years of follow-up were modelled as a linear function of the cumulative activity of ^131^I administered 2 years or more before. Radioiodine activity was also treated as a categorised variable, reflecting 1–4 standard treatments of 3.7 GBq. This could not be done for leukaemias due to insufficient numbers.

The significance of the parameters was tested by comparing the module of EPICURE deviance of nested models. The analysis was done by using AMFIT software (Preston *et al*, 1991). Confidence intervals (95% CI) of the risk were estimated using maximum likelihood methods (Moolgavkar and Venzon, 1987). When convergence was not obtained and lower or upper bounds not estimable with this technique, a question mark was reported in the results. All analyses of the pooled cohort were stratified on the study groups and heterogeneity between study groups was sought.

## RESULTS

The study concerned 6841 thyroid cancer patients diagnosed during the period 1934 and 1995 (Table 1

**Table 1:** Characteristics of the patients treated for a papillary or a follicular thyroid cancer of the three cohorts and of the pooled cohort

	**Swedish cohort**	**Italian cohort**	**French cohort**	**Pooled cohort**
*Patients*				
Number	1894	1894	3053	6841
Treatment period (years)	1951–1977	1958–1995	1934–1995	1934–1995
Males (%)	460 (24)	433 (23)	688 (23)	1581 (23)
Mean age at thyroid cancer diagnosis (range)	49 (5–91)	44 (5–82)	42 (2–91)	44 (2–91)
Mean follow-up duration (years)	20 (2–46)	8 (2–35)	12 (2–55)	13 (2–55)

*Treatment*				
Thyroid surgery (%)	1783 (94)	1891 (100)	3002 (98)	6676 (98)
External beam radiotherapy (%)	725 (38)	17 (1)	452 (15)	1194 (17)
Treatment with ^131^I (%)	795 (42)	1576 (83)	1854 (61)	4225 (62)
Mean cumulative ^131^I activity in GBq^a^ (range)	3.8 (0.4–49.2)	5.9 (0.9–34.4)	6.9 (0.2–55.5)	6.0 (0.2–55.5)

a In case of treatment with ^131^I, cumulative activity administered up to 2 years before the end of follow-up or the diagnosis of second primary malignancies is measured.

). About 20% of them were lost to follow-up. An approximate 3 : 1, female to male ratio was seen and the mean age at diagnosis was 44 years. In all, 17% were treated with external radiotherapy and 62% received ^131^I. Only 9% received both external radiotherapy and ^131^I. The mean follow-up period after thyroid cancer diagnosis was 13 years (range: 2–55 years) (Table 1). The mean interval of time between thyroid cancer diagnosis and SPM was 15 years (range: 2–55 years), and the mean age at SPM diagnosis was 64 years (range: 21–99 years; data not shown).

In total, 576 patients were diagnosed with an SPM, among whom 13 developed a third malignant neoplasm less than 2 years after the SPM. For all the 6841 thyroid cancer patients, an overall significantly increased risk of cancer of 27% (95% CI: 15–40) compared to the general population of each of the three countries was seen and the risk did not differ between men and women (data not shown). Significantly increased risk of cancer in the digestive tract (SIR=1.2, *n*=126), bone and soft tissue (SIR=5.9, *n*=19), skin melanoma (SIR=2.3, *n*=25), kidney (SIR=2.6, *n*=31), central nervous system (SIR=2.1, *n*=21), endocrine glands other than thyroid (SIR=3.6, *n*=18), and leukaemias (SIR=1.8, *n*=18) was seen for the 6841 patients. No significant gender difference was seen (data not shown). Furthermore, a significantly increased risk was seen for female breast cancer (SIR=1.3, *n*=128) and for genital male cancers (SIR=1.5, *n*=30).

No significant association was found between exposure to external radiotherapy and risk of SPM, except for bone and soft-tissue cancers (RR=2.9, 95% CI: 1.2–7.3). Furthermore, no interaction was evidenced between external radiotherapy and ^131^I administration for the risk of SPM.

The risk of SPM occurrence in relation to ^131^I administration is described in Table 2

**Table 2:** Observed number of SPMs, standardized incidence ratio (95% confidence interval) and risk of SPM in relation to ^131^I administration, among the 6841 patients treated for a papillary or follicular thyroid cancer

	**All the patients (PYR=77 955)**			**^131^I therapy (PYR=37 702)**			**No ^131^I therapy (PYR=40 253)**			
**Cancer site (ICD9 code)**	**Number of SPMs**	**SIR^a^**	**(95% CI)**	**Number of SPMs**	**SIR^a^**	**(95% CI)**	**Number of SPMs**	**SIR^a^**	**(95% CI)**	**Relative risk^b^: ^131^I *vs* no ^131^I (95% CI)**
Oral cavity (140–145)	13	2.0	(1.0–3.4)	10	2.6	(1.2–4.8)	3	0.8	(0.2–2.2)	2.8 (0.8–13.0)
Salivary glands (142)^c^	7	–		6	–		1	–		7.5 (1.2–143)
Pharynx (146–149)	3	0.5	(0.09–1.6)	1	0.3	(0.02–1.5)	2	0.9	(0.2–2.9)	0.4 (0.02–4.7)
Digestive tract (150–159)	126	1.3	(1.0–1.5)	61	1.2	(0.9–1.5)	65	1.4	(1.0–1.8)	1.1 (0.8–1.5)
Stomach (151)	20	1.1	(0.6–1.7)	10	1.0	(0.5–1.7)	10	1.0	(0.5–1.7)	1.3 (0.5–3.2)
Colon and rectum (153–154)	69	1.3	(0.9–1.6)	37	1.4	(0.9–1.9)	32	1.1	(0.7–1.7)	1.3 (0.8–2.0)
Respiratory organs (161–163)	37	0.9	(0.6–1.3)	20	1.0	(0.6–1.6)	17	0.8	(0.4–1.4)	1.1 (0.5–2.3)
Lung cancer (162)	32	1.0	(0.6–1.4)	18	1.0	(0.6–1.6)	14	0.9	(0.4–1.7)	1.1 (0.5–2.3)
Bone and soft tissue (170–171)	19	5.9	(3.6–9.0)	14	5.8	(2.5–11.2)	5	1.8	(0.3–5.5)	4.0 (1.5–12.4)
Skin melanoma (172)	25	2.5	(1.6–3.7)	11	2.1	(1.1–3.8)	14	2.9	(1.6–4.9)	0.8 (0.3–1.8)
Breast (174)	128	1.3	(1.0–1.5)	54	1.2	(0.9–1.6)	74	1.3	(1.0–1.7)	0.8 (0.5–1.1)
Female genital organs (179–183)	57	0.7	(0.5–1.0)	36	0.9	(0.6–1.3)	21	0.6	(0.3–0.9)	2.2 (1.3–3.9)
Uterus (179–182)	39	1.0	(0.7–1.4)	25	1.1	(0.7–1.7)	14	0.8	(0.4–1.4)	2.3 (1.2–4.7)
Ovary (183)	20	0.5	(0.3–0.8)	12	0.7	(0.3–1.2)	8	0.4	(0.1–0.8)	2.0 (0.8–5.2)
Male genital organs (185–186)	30	1.6	(1.0–2.4)	16	1.3	(0.6–2.3)	14	2.0	(1.1–3.4)	1.1 (0.5–2.3)
Urinary tract (188–189)	50	1.8	(1.3–2.4)	31	2.1	(1.4–3.1)	19	1.4	(0.7–2.3)	1.5 (0.9–2.8)
Bladder (188)	19	1.2	(0.7–1.9)	12	1.4	(0.7–2.3)	7	0.4	(0.1–1.2)	1.6 (0.6–4.5)
Kidney (189)	31	2.6	(1.7–3.8)	19	2.6	(1.5–4.4)	12	2.6	(1.3–4.5)	1.5 (0.7–3.3)
Central nervous system (191–192)	21	2.5	(1.5–3.8)	13	3.0	(1.6–5.2)	8	1.9	(0.8–3.7)	2.2 (0.9–5.7)
Endocrine glands (194)	18	3.1	(1.6–5.3)	10	5.2	(2.4–9.7)	8	1.5	(0.4–1.4)	1.6 (0.6–4.3)
Lymphoma (200–202)	17	1.1	(0.6–1.8)	10	1.1	(0.5–2.2)	8	1.2	(0.5–2.5)	1.0 (0.4–2.8)
Multiple myeloma (203)	6	1.4	(0.5–2.9)	4	1.6	(0.5–3.6)	2	0.6	(0.03–2.6)	1.4 (0.3–0.7)
Leukaemia (204–208)^d^	18	1.6	(0.8–2.7)	12	1.9	(0.8–3.6)	6	1.2	(0.4–2.8)	2.5 (1.0–7.4)
Other^e^	21	0.9	(0.5–1.5)	8	0.6	(0.2–1.3)	13	1.2	(0.6–2.1)	0.6 (0.2–1.5)
At least one cancer^f^	576	1.3	(1.2–1.4)	301	1.3	(1.1–1.5)	275	1.3	(1.1–1.4)	1.2 (1.0–1.4)

PYR=Number of person-years of follow-up.

a Standardised incidence ratio adjusted on external radiotherapy.

b Relative risk stratified on study group and adjusted on external radiotherapy.

c No SIR could be calculated for this cancer since French reference rates are lacking for this localization.

d One woman developed a leukaemia less than 1 year after the occurrence of a breast cancer.

e Including the following sites (ICD9 code): 160, 164, 165, 175, 184, 187, 190, 195–199.

f Number of patients with at least one cancer excluding thyroid cancers and nonmelanoma skin cancers: 13 patients with two second malignancies within 2 years.

. When contrasting those exposed and not exposed to ^131^I, after stratifying for study cohort and adjusting for external radiotherapy, increased relative risks were seen for the bone and soft tissue (RR=4.0), female genital organs (RR=2.2), central nervous system (RR=2.2), and leukaemia (RR=2.5) (Table 2). Of the 13 cancers of oral cavity, seven were cancers of the salivary glands: six of them occurred in patients treated with ^131^I (*n*=4225), and only one in patients treated by other means (*n*=2616). SIR was not calculated for salivary gland cancer since French reference rates are lacking for this localisation but analysis using internal comparison yielded a relative risk of 7.5 (95% CI: 1.2–143).

Among the 344 patients aged less than 20 years at thyroid cancer diagnosis, 13 SMNs occurred, including two digestive tract, one lung, two bone, five breast, two endocrine cancers, and one malignant lymphoma. Compared to the general population, the overall risk of SPM was significantly increased (SIR=2.5), as well as the risk of secondary breast cancer (SIR=3.4). In all, 61% of the young patients were treated by ^131^I and no carcinogenic effect of ^131^I was found (RR=1.1).

An increased risk of both solid tumours and leukaemias was seen with increasing cumulative activity of ^131^I administered after adjustment for external radiotherapy (Table 3

**Table 3:** Risk of SPMs as a function of the cumulative ^131^I activity administered 2 years or more before the diagnosis of SPM

		**No external radiotherapy**		**External radiotherapy**		**All patients**	
**Type of SPM**	**Amount of ^131^I (GBq)**	**Number of SPM/PYR**	**RR^a^ (95% CI)**	**Number of SPM/PYR**	**RR^a^ (95% CI)**	**Number of SPM/PYR**	**RR^b^ (95% CI)**
*Solid cancers*^c^	⩽0.2	186/29625	1 (ref)	84/10629	1 (ref)	270/40254	1 (ref)
	[0.2–3.6]	98/10795	1.2 (0.9–1.5)	23/2553	1.1 (0.7–1.7)	121/13348	1.2 (0.9–1.4)
	[3.7–7.3]	78/14330	0.9 (0.7–1.2)	24/2178	1.4 (0.9–2.3)	102/16508	1.0 (0.8–1.3)
	[7.4–14.7]	32/4693	1.4 (1.0–2.1)	12/1184	1.6 (0.9–3.0)	47/5877	1.5 (1.0–2.0)
	⩾14.8	12/1475	1.5 (0.8–2.6)	7/495	2.1 (0.9–4.7)	19/1970	1.6 (1.0–2.6)
							
*Leukaemias*	⩽0.2	4/29625	1 (ref)	2/10629	1 (ref)	6/40254	1 (ref)
	[0.2–3.6]	3/10795	1.4 (0.3–6.6)	1/2553	2.2 (0.1–23.5)	4/13348	1.8 (0.4–6.3)
	[3.7–18.4]	4/19644	2.6 (0.5–11.8)	3/3485	4.3 (0.7–34.9)	7/23129	3.1 (1.0–10.3)
	⩾18.5	0/853	–	1/372	14.0 (0.6–167.4)	1/1225	7.5 (0.4–48.7)

PYR=Number of person-years of follow-up.

a Relative risks stratified on study group.

b Relative risks adjusted on external radiotherapy and stratified on study group.

c All cancers excluding leukaemias, thyroid cancers, and nonmelanoma skin cancers.

). A linear relationship without any significant quadratic effect best fitted the description of the solid tumours (*χ*^2^=0.46, *P*=0.5). An ERR of 3.5 % (95% CI: 0.9–6.9%) per GBq of ^131^I was seen. The ERR was similar for patients who did or did not receive external radiotherapy, being 3.4% (95% CI: 0.2–7.8%) and 3.5% (95% CI: 0.01–8.6%), respectively (Table 4

**Table 4:** Excess of relative risk per cumulative activity of ^131^I in GBq (ERR) according to external radiotherapy for major types of SPMs

	**No external radiotherapy**		**External radiotherapy**		**All the patients**		
**Type of SPM**	**ERR per GBq of ^131^I^a^ (95% CI)**	**Test of trend^b^**	**ERR per GBq of ^131^I^a^ (95% CI)**	**Test of trend^b^**	**ERR per GBq of ^131^I^c^ (95% CI)**	**Test of trend^b^**	**Test of heterogeneity^d^**
Solid cancers^e^	0.03 (0.002–0.08)	0.03	0.04 (0.00006–0.09)	0.05	0.04 (0.009–0.07)	<0.01	0.6
Soft-tissue and bone cancer	0.29 (?–1.74)^f^	0.2	1.04 (?–3.93)^f^	<0.001	0.61 (?–2.41)^f^	<0.001	0.9
Colorectal cancer	0.15 (0.02–0.38)	0.01	0.02 (?–0.20)^f^	0.7	0.10 (0.08–0.27)	0.03	0.4
Breast cancer	0.002 (?–0.07)^f^	1.0	−0.02 (?–0.04)^f^	0.3	−0.01 (?–0.04)^f^	0.6	0.3
Leukaemias	0.22 (?–1.30)^f^	0.2	0.59 (?–2.34)^f^	<0.01	0.39 (?–1.54)^f^	0.01	0.4

a Stratified on study group.

b Test of trend for a linear dose–effect relationship: *P*-value.

c Adjusted on external radiotherapy and stratified on study group.

d Test of heterogeneity of the ERR between patients exposed and nonexposed to external radiotherapy.

e All cancers except leukaemias, thyroid cancers, and nonmelanoma skin cancers.

f Lower bound not calculable.

). An EAR of 14.4 solid cancers per GBq of ^131^I administered and per 10^5^ person-years of follow-up was estimated. No heterogeneity of the results was found between cohorts (*P*=0.7). An even stronger relationship was found between the cumulative activity of ^131^I administered and the risk of leukaemia (Tables 3 and 4). The relationship was linear, without any significant quadratic effect (*χ*^2^=0.2, *P*=0.7). The ERR for leukaemia was 39% (95% CI: ?–154%) per GBq and the EAR was estimated to be 0.8 cases per GBq of ^131^I administered and per 10^5^ person-years of follow-up. External radiotherapy, treated as a binary variable (exposed *vs* nonexposed), did not influence significantly the relationship between the amount of ^131^I administered and the risk of leukaemia.

A significant dose–effect relationship was found for bone and soft-tissue cancers and for colorectal cancers after adjustment for external radiotherapy (Tables 4 and 5

**Table 5:** Relative risk of soft-tissue and bone cancer, colorectal cancer, and breast cancer as a function of the cumulative ^131^I activity administered 2 years or more before the diagnosis of SPMs

	**Number of SPMs and RR^a^ (95% CI)**					
**Amount of ^131^I (GBq)**	**Soft-tissue and bone cancer**		**Colorectal cancer**		**Breast cancer**	
⩽0.2	5	1 (ref)	32	1 (ref)	74	1 (ref)
[0.2–3.6]	5	2.9 (0.8–10.7)	12	0.9 (0.5–1.8)	23	0.9 (0.5–1.4)
[3.7–7.3]	4	3.4 (0.8–13.3)	13	1.1 (0.6–2.2)	19	0.6 (0.3–1.0)
[7.4–14.7]	3	7.7 (1.5–33.3)	8	2.5 (1.0–5.5)	9	1.0 (0.4–1.9)
⩾14.8	2	13.0 (1.7–67.1)	4	3.2 (0.9–8.7)	3	0.9 (0.2–2.5)

a Relative risk adjusted on external radiotherapy and stratified on study group.

), the ERR per GBq of ^131^I administered being 61% (95% CI: ?–241%, *P*<0.01) and 10% (95% CI: 1–27%, *P*=0.03), respectively, with no evidence for an interaction between ^131^I administration and external radiotherapy. A significant relationship was found between the risk of female genital cancer and the amount of ^131^I administered, although there was no significant increase in the overall SIR. For cancer of the central nervous system, a nearly significant increase of the ERR with the amount of ^131^I administered was found (*P*=0.13).

The ERR for solid tumours did not vary widely with time after exposure to ^131^I: among the 3211 patients followed at least 10 years after thyroid cancer treatment, the ERR of SPM more than 10 years after the last ^131^I treatment was 6% (95% CI: 1–12%) per GBq of ^131^I administered.

## DISCUSSION

We found a significant 30% increased risk of developing a SPM in one of the largest cohorts of thyroid cancer patients reported to date. A linear dose–response relationship with ^131^I administration was seen for all cancers combined and for leukaemia, and we estimated that a treatment of 3.7 GBq of ^131^I will induce an excess of 53 solid malignant tumours and 3 leukaemias, in 10 000 patients during 10 years of follow-up. In addition, we identified a strong relationship between the cumulative activity of ^131^I and risk of bone and soft-tissue cancer, colorectal cancer, and salivary gland cancer. The increased incidence of SPM seen in the present study and previously reported (Tucker *et al*, 1985; Hall *et al*, 1990, 1991; Dottorini *et al*, 1995; Edhemovic *et al*, 1998) could also be related to an increased medical surveillance, common aetiological factors including hereditary factors, or misclassified metastases. Although increased medical surveillance could contribute to an earlier detection of cancers with a long latency, it could hardly explain the excess of leukaemia and of solid cancers with a poor prognosis and a rapid evolution.

Ionising radiation is the only established cause of thyroid cancer in humans (UNSCEAR, 2000). Other risk factors, such as diet, reproductive factors, deficiency or excess of iodine, changes in height and weight, have been involved in some studies (La Vecchia *et al*, 1999; Negri *et al*, 1999; Dal Maso *et al*, 2000; Horn-Ross *et al*, 2001). It is not likely that any of these factors would profoundly influence the risk related to radioiodine since we found a difference between exposed and nonexposed tumours, and, for some tumours, a dose–response relationship. Some rare familial syndromes associated with an excess risk of thyroid cancer include familial adenomatous polyposis, Carney complex, and Cowden syndrome (Stratakis *et al*, 1997; Lindor and Greene, 1998). Owing to their rarity, these syndromes probably did not influence our risk estimates.

In all, 20% of patients were lost to follow-up before 1997 as a result of the routine long-term follow-up programmes. This has probably not introduced any bias in the study, because the frequency of patients who lost to follow-up was quite similar among patients who did or did not receive ^131^I treatment. Moreover, if the end point of the study is fixed at 31 December 1992, a similar dose–effect relationship is found, with an ERR of 3.3% per GBq of ^131^I (95% CI: 0.2–7.5%), whereas about 10% of patients are lost to follow-up.

All efforts were made to increase the specificity of SPM through exclusion of distant metastases particularly in the lungs, bones, and brain. When excluding the sites where possible misclassification of SPM could occur, the overall risk of SPM remained significantly increased in the ^131^I exposed group (*P*=0.02).

Despite the known carcinogenic effect of ionising radiation, no association was evidenced between external radiotherapy and SPM occurrence in our study except for soft and bone cancers, and no interaction was found between external radiotherapy and ^131^I administration. This could result from the small number of patients treated by external radiotherapy: less than 1/5 of the patients were treated by external radiotherapy and less than 1/10 received external radiotherapy and ^131^I.

All the analyses were nevertheless adjusted on external radiotherapy in an attempt to access the own effect of ^131^I on SPM occurrence.

The increased risk of leukaemia was previously observed in the Swedish cohort, but not in the Italian and French cohorts, demonstrating the advantage of a pooled analysis. The dose–response relationship was clearly linear, and most cases of leukaemia occurred in patients treated with high cumulative activities of ^131^I. We also evidenced a significant relationship between the cumulative activity of ^131^I administered and the risk of solid cancer, with an excess of 4% per GBq. This major result was already suggested in each of the three cohorts included in our pooled analysis.

Significantly increased risks with higher cumulative amount of ^131^I was only seen for colorectal cancer, bone and soft-tissue cancer, and salivary gland cancer. The excess of colorectal cancer was previously evidenced only in the French cohort (de Vathaire *et al*, 1997), and the excess of salivary gland cancer was found in the Swedish (Hall *et al*, 1991) and Italian (Dottorini *et al*, 1995) cohorts. In contrast, the excess of bone and soft-tissue cancers following ^131^I administration has not been previously reported. In fact, an excess of these cancers could only be shown in large cohorts, because they are infrequent in the general population. These cancers have been frequently associated to local high doses of radiation delivered by external radiotherapy (UNSCEAR, 2000); indeed, we found that they were associated with exposure to external radiotherapy. However, the increasing risk of soft tissue and bone cancer with a higher cumulative amount of ^131^I remained significant after adjustment on exposure to external radiation therapy. Finally, the dose–response relationship seen in our series may be due to the use of larger activities of ^131^I in patients with bone metastases, and in these patients a dedifferentiation of metastatic lesions may occur and can rarely be totally separated from a SPM. However, the rarity of this event argues against its large influence on our risk estimate.

Ionising radiation can induce tumours of the central nervous system, although the relationship is weaker than for many other tumours, and most radiation-associated tumours are benign (UNSCEAR, 2000). The increased risk of central nervous system cancers in our study leads to question about the radiation doses delivered to the brain after repeated ^131^I administrations for thyroid cancer.

An increased incidence of breast cancer was found among women treated for thyroid cancer as compared to the general population; however, this was not related to ^131^I exposure, even among the women who were less than 40 years old at the time of thyroid cancer diagnosis. This confirms that the radiation dose to the mammary tissue is low following ^131^I treatment, despite the expression of the sodium iodide symporter in some physiological or pathological conditions (Tazebay *et al*, 2000); in fact, the mammary tissue is rarely visualised on ^131^I total body scanning (Hammami and Bakheet, 1996). This finding is in accordance with a case–control study nested in the Swedish cohort (Hall *et al*, 1992) and may be related to a closer medical surveillance or to common aetiological factors. In fact, some population-based cohort studies indicate an increase of thyroid cancer after breast cancer treatment and an increase of breast cancer after thyroid cancer (Ron *et al*, 1984; Teppo *et al*, 1985; Li *et al*, 2000). However, other studies only identified an increased risk of breast cancer after thyroid cancer treatment (Vassilopoulou-Sellin *et al*, 1999; Chen *et al*, 2001).

Similarly, an increased incidence of kidney cancer was found but was not related to ^131^I exposure. This is in accordance with a previous case–control study nested in the Swedish cohort, and is not surprising as kidney cancer is not described as a frequently radiation-induced cancer (UNSCEAR, 2000). All population-based cohort studies of patients treated for thyroid cancer reported an increased incidence of kidney cancer (Osterlind *et al*, 1985; Tucker *et al*, 1985; Shikhani *et al*, 1986; Akslen and Glattre, 1992; Ishikawa *et al*, 1996; Edhemovic *et al*, 1998), which may be related to common etiological factors.

For each activity of ^131^I administered, the organ doses delivered by ^131^I to thyroid cancer patients are higher than in euthyroid subjects due to a decreased renal clearance and prolonged body retention of ^131^I caused by the hypothyroid condition (M'Kacher *et al*, 1996). Due to the local accumulation of ^131^I, the stomach, salivary glands, and bladder receive the highest radiation doses. In hypothyroid patients, ^131^I is also accumulated in the colon lumen due to a decreased colonic motility. The risk of colorectal and salivary gland cancers should be minimised by a simple routine of having patients drink large quantities of fluids and lemon juice, and by the use of laxatives. Despite the known accumulation of ^131^I in the stomach and bladder, we did not find any increased risk for these sites.

Our results concern high activities of ^131^I and do not apply to the general population or to patients treated with ^131^I for hyperthyroidism, since the accumulation of ^131^I is low or absent in the colon lumen of euthyroid or hyperthyroid patients.

In conclusion, we report an excess of 53 cases of solid malignancy and of three cases of leukaemia per 10 years among 10 000 patients treated with a standard activity of 3.7 GBq of ^131^I. Indeed, these results do not contraindicate the therapeutic use of ^131^I in patients for whom a clinical benefit is expected. However, as the dose–response relationships for ^131^I administration were linear, it seems necessary to restrict the repeated use of ^131^I to thyroid cancer patients in whom it may be beneficial (Schlumberger, 1998).
